# DOES SELF-REPORTED DISCRIMINATION INCREASE AS COGNITION DECLINES?

**DOI:** 10.1093/geroni/igad104.2701

**Published:** 2023-12-21

**Authors:** Douglas Hanes, Sean Clouston

**Affiliations:** Stony Brook University, Stony Brook, New York, United States; Stony Brook University, Stony Brook, New York, United States

## Abstract

Progressing cognitive decline causes limitations on daily life and emotional changes, including increased aggression, agitation, and restlessness that might occasion greater scrutiny, limitations, discrimination, and disrespect due to a combination of care-provision and ageism. While there are a number of studies of discrimination as a cause of cognitive decline, there are no studies of how real and perceived discrimination during cognitive decline might affect the aging person and their caregivers. This study analyzes whether people currently experiencing cognitive decline report greater frequency of discrimination. Using the Health and Retirement Study (HRS; 2006–18; N = 37,059), we used multilevel ordered logistic regression to test the role of changes in cognitive performance in predicting self-reported frequency of six forms of discrimination, adjusting for age, socioeconomic, and demographic covariates. We found that lower cognitive scores were associated with increased discrimination frequency in three forms of treatment: less respect (β=-0.010; p=0.022), being treated as “not smart” (β=-0.026; p< 0.001), and worse medical treatment (β=-0.014; p=0.027) though no differences were evident in service quality (β=-0.002, p=0.736), evidence of being threatened (β=-0.11, p=0.069), or having people report being afraid of you (β=-0.004, p=0.455). Results suggest that perceptions of discrimination could emerge as a result of declining cognitive performance and highlight the need for greater sensitivity to discrimination among older adults in both everyday and medical settings. Further investigation will enable us to distinguish between experienced discrimination due to changes in treatment versus ageism.

